# Factors influencing consumption of traditional diets: stakeholder views regarding sago consumption among the indigenous peoples of West Papua

**DOI:** 10.1186/s40066-022-00390-5

**Published:** 2022-10-07

**Authors:** Fathir Fajar Sidiq, David Coles, Carmen Hubbard, Beth Clark, Lynn J. Frewer

**Affiliations:** School of Natural and Environmental Sciences, Newcastle University, Newcastle Upon Tyne, NE1 7RU UK

**Keywords:** Sago, Food security, Traditional diets, Indigenous peoples, West Papua

## Abstract

**Background:**

The indigenous people living in the province of West Papua may experience potential food insecurity, in part attributable to increased local adoption of, and reliance on, imported foods such as rice. At the same time, the consumption of sago, a traditional local food, is lower than other types of carbohydrate foods such as wheat and cassava. Various factors may act as influential drivers of local diets and related agricultural practices, such as local socio-economic and agronomic factors, as well as cultural practices which in turn may be influenced by the attitudes and opinions of stakeholders with interests in the supply chain.

**Methods:**

Qualitative methodology (semi-structured interviews) was applied to understand the various factors perceived by a selected number of stakeholders that influence sago consumption among the indigenous peoples of West Papua. These stakeholders included politicians, local and national civil servants, academics, sago farmers, and food activists (*n *= 18). Thematic analysis was used to analyse the data.

**Results:**

The results indicate that the stakeholders perceive that the majority of the West Papuan people regard sago as a traditional food that is critical to, and inseparable from, local culture and food production practices. The results suggest that the stakeholders interviewed support the consumption of sago to improve food security for the indigenous people in West Papua, with a need for future policy to be developed to support this.

**Conclusions:**

The evidence presented here suggests a diverse range of local stakeholders support the continuation of sago production and consumption, both from the perspective of improved food security, and in order to conserve cultural associations and activities within local communities. According to the stakeholders interviewed, this can be best achieved through improved engagement of local communities with sago production policies, innovation in current practices, and agronomic management. Local policies should be developed to ensure that sago remains an integral component of the Papuan people's culture, and develops into a significant commodity with economic value which simultaneously contributes to environmental targets.

**Supplementary Information:**

The online version contains supplementary material available at 10.1186/s40066-022-00390-5.

## Introduction

Food insecurity remains a global problem, with 690 million people being malnourished even before Covid-19 disrupted supply chains [1]. The current global crisis in the supply of wheat due to the conflict in Ukraine [2], shows just how fragile food security can be and points to the importance of a reliable and resilient local food source that is independent of circumstances elsewhere. A significant concern relates to food insecurity in indigenous populations, where indigenous people are defined as those who retain the knowledge of the land and associated food resources [3]. Indigenous peoples comprise approximately 5% of the world’s population, at the same time accounting for 15% of the poorest people on earth [4]. Food insecurity in these groups is exacerbated by the “nutrition transition” [5] from the use of traditional diets relying on local resourcing to the consumption of westernised diets, which are less healthy and more energy-dense, with high contents of sugar, salt, and saturated fats [6]. The consumption of these foods is increasing due to indigenous people’s preferences for the convenience of modern foods [7, 8]. In addition, reliance on imported foods increases the vulnerability of local supply chains to system shocks caused by climate change or geopolitical turbulence [9]. Traditional diets may also be repositories of cultural knowledge or form the basis of biodiverse, local ecological systems [9]. This suggests that increased reliance on local, traditional food production systems will be associated with multiple health and environmental advantages concerning the promotion of food security. Consequently, it is important to understand the barriers to, and facilitators of, the consumption of traditional diets by indigenous peoples.

Stakeholders with interests in food security (e.g. policymakers, educators and extension specialists, members of civil society groups, and those within the food industry) play an important role in shaping access to healthy and sustainable diets [10]. Hence, stakeholders’ views on what constitutes healthy practices and the role of traditional foods in promoting food security are important, as they will shape food availability and hence consumers’ food choices in the supply chain [10]. Stakeholders’ perspectives are important as one of the approaches applied to the development of community empowerment where stakeholders have different roles and responsibilities [11]. This research aims to assess the views of different, potentially influential stakeholders on the adoption or otherwise of traditional food, sago, in the diets of the indigenous people in West Papua. A case study approach was adopted, which provides information about the potential factors that motivate local consumers to adopt sago in their diets. The case study approach adopted also enables the identification of other facilitators and barriers to improved local food security. The results will contribute to improved food security policies in the region.

The research was conducted in Tanah Papua (Land of Papua), which comprises two Indonesian provinces: Papua and West Papua. Both provinces account for more than half of Indonesia's total biodiversity, particularly endemic flora and fauna [12]. Tanah Papua's cultural diversity comprises at least 250 indigenous ethnic groups, each with its own language and rich traditional ecological knowledge [13]. The Food Security and Vulnerability Atlas (FSVA), published by the Food Security Agency of the Ministry of Agriculture of the Republic of Indonesia [14], indicates that some regions of Indonesia are food insecure, including the Papua and West Papua provinces, despite the availability of traditional foods to local peoples. This is partly because of increased local adoption of, and reliance on, imported foods such as rice [15]. Sago, a traditionally produced local food (*Metroxylon sagu* Rottb.), has the potential to reduce reliance on imported grains such as rice within local communities, improve the sustainability of locally produced foods, and contribute to the preservation of local ecosystems and the ecosystem services which they deliver [15]. The sago palm (*Metroxylon sagu* Rottb) is found in the tropical lowland forest and freshwater wetlands across Southeast Asia and New Guinea. It is one of the oldest crops and was the staple food in those areas. It is the primary source of sago, which is the food derived from it. In general, sago is a food derived from sago palm, which is the plant which can be described as the originating crop [16, 17]. Indonesia has the world's largest sago palm plantations, with around 1.1 million hectares (ha) of sago palm, or 51.3% of the global total of about 32 million ha [18]. Around 90% of Indonesian sago is grown in Papua and Maluku [19]. Official data [20] show that there is 5.4 million ha of Indonesian sago forests, with more than 95% in the Papua region. Sago production capacity in the provinces of Papua and West Papua is projected to be over 13 million tonnes of dried starch per year. Sago starch yield per unit area is potentially about 3 to 4 times higher than that of rice, corn, or wheat, and about 17 times higher than that of cassava [21]. However, only less than half a percent is utilised, and an estimated six million tonnes of dried starch is not used in diets due to inadequate harvesting [22]. Due to the sago distribution chain’s inefficiency and lack of market awareness, sago farmers have a poor bargaining position [23]. At the same time, smallholder farmers are gradually shifting from sago production to oil palm plantations that attract higher returns [24].

Sago consumption by local consumers is very low in comparison to other commodities. Sago is consumed at a rate of 0.36 kg per capita per year. In comparison rice is consumed at a rate of 97.1 kg per capita per year, and wheat is consumed at a rate of 18.2 kg per capita per year [14]. Only 1.8% of Indonesians consume sago. Sago is less popular than cassava (19.6%) and wheat flour (30.2%) [25]. Food diversification is considered an appropriate approach to ensure national food security. Artificial^1^ rice production using local natural resources may help to provide food security, using sago as one of the main ingredients [26].

Research into local production has focused on the attitudes and priorities of farmers who directly produce sago, which indicated that diversification of sago products within this supply chain has beneficial economic consequences for primary producers [27]. There is a research gap regarding the opinions of other supply chain actors and interested stakeholders in West Papua. Hence, the aims of the research were to investigate the various factors perceived by a selected number of stakeholders that influence sago consumption among the indigenous peoples of West Papua. This research aims to assess the views of different, potentially influential stakeholders on the adoption or otherwise of traditional food, sago, in the diets of the indigenous people in West Papua, as these stakeholders will directly shape the sago supply chain (see also, inter alia [28]). While the authors recognise that broader community members not involved in the sago food chain other than via consumption of products are an important stakeholder at the end of the value chain, and as a driver of demand, this is beyond the scope of the present study and will be investigated in future research using survey methodology. The views of stakeholders were solicited regarding:

- What are the factors that motivate the indigenous peoples of West Papua to produce and/or consume sago?
- Are strategies needed to increase Papuans’ sago consumption? If yes, what form might these take?
- What are the barriers to producing and consuming sago?
- What are the stakeholders understandings of sago eating culture?
- What are the implications for future sago policies regarding sago production and consumption in West Papua?

## Methods

### Study design

A qualitative approach was applied to understanding the factors influencing sago consumption among the indigenous peoples of West Papua from the perspective of various stakeholders: politicians, local and national civil servants, academics, sago farmers, and food activists. The perspectives of consumers will be evaluated in a separate study in relation to stakeholder views.

Semi-structured interviews were used to investigate stakeholder’s perspectives on sago production and consumption behaviour among indigenous peoples of West Papua. This will provide an in-depth understanding of the viewpoints, experiences, and practices of important stakeholders regarding definition and identification of research gaps. Examining the perspectives of various key stakeholders will ensure that the issue is not viewed through a single lens [29]. The consolidated criteria for reporting qualitative research (COREQ) checklist (Additional file 1) for interviews was used to guide reporting [30]. This study adopted the Global Code of Conduct for Research in Resource-Poor Settings from the four TRUST values: *fairness, respect, care*, and *honesty* [31]. These four values were the foundation throughout this research process.

The lead author contacted several gatekeepers^2^ who assisted the author at the case study site. The role of the gatekeeper was to access the appropriate key informants. The research questions were provided to gatekeepers in draft form for further co-design. Ethical approval was obtained from the University of Newcastle, UK ethics committee, Project #: 19-SI-014, on the 28th of February 2019, prior to data collection.

### Research setting

The research was conducted in Sorong Selatan Regency, West Papua Province, Indonesia. Sorong Selatan Regency is located between 01°00ʹ–02°30ʹ North Latitude and 131°00ʹ–133°00ʹ East Latitude. The total area of Sorong Selatan Regency is 8424.165 km^2^, consists of a land area of 6891.551 km^2^ (95.1%) and an ocean area is 1532.614 km^2^ (4.9%). The topography of most areas of this regency is a valley [32]. Data were collected in four districts, namely Teminabuan, Matemani, Saifi, and Seremuk. Data collection was also carried out in Jakarta, Bogor, and Yogyakarta via Zoom calls. The total area of the sago plantation is 311,591 ha or 45% of the total area of Sorong Selatan Regency [33].

### Participant sampling and recruitment

A purposive recruitment of politicians (*n *= 1; 5.6%), sago farmers (*n = *5; 27.8%), academics (*n *= 2; 11.1%), and local civil servants (*n *= 5; 27.8%) was conducted by the gatekeepers. The lead author recruited the national civil servants (n = 3; 16.7%) and food activists (*n *= 2; 11.1%). These stakeholders were chosen to ensure that all various interests in the management of sago in South Sorong, West Papua were covered. Academics who act as innovators, local and national governments as facilitators, and activist groups were included as potential policy implementers [11]. Eighteen participants (*n *= 18) were interviewed in total. *N* = 18 is an appropriate size for such a stakeholder analysis, where there is a requirement to investigate local expert knowledge in an in-depth way, rather than solicit opinions from a much larger area, but including participants with less local agronomic and cultural expertise [34, 35]. Furthermore, previous research recommended that qualitative studies require a minimum sample size of at least 12 to reach data saturation [36, 37]. Therefore, a sample of 18 was deemed sufficient for the thematic analysis of this research. During the data collection process, the gatekeeper facilitated identification of, and access to, the key informants. Participant information is summarised in Table 1.

**Table 1:** List of participants

Participant	Position	Roles	Gender	Location
#01	Politician	Policy maker	Male	Teminabuan
#02	Local civil servant	District leader	Male	Teminabuan
#03	Local civil servant	District leader	Male	Matemani
#04	Local civil servant	District leader	Male	Matemani
#05	Local civil servant	Food security officer	Male	Teminabuan
#06	Local civil servant	Food security officer	Female	Teminabuan
#07	Academia	Lecturer	Female	Teminabuan
#08	Academia	Sago researcher	Male	Bogor
#09	Farmer	Sago farmer	Female	Saifi
#10	Farmer	Sago farmer	Male	Saifi
#11	Farmer	Sago farmer	Male	Seremuk
#12	Farmer	Sago farmer	Male	Seremuk
#13	Farmer	Sago farmer	Male	Teminabuan
#14	National civil servant	Special autonomy officer	Male	Jakarta
#15	National civil servant	Food security officer	Male	Jakarta
#16	National civil servant	Coordinating Ministry of Economic Affairs	Male	Jakarta
#17	Food activist	Sago enthusiast	Male	Bogor
#18	Food activist	Sago enthusiast	Male	Yogyakarta

Female stakeholders are underrepresented in the stakeholder sample (3 of 18 participants), which represented academia, sago farming, and the local civil service. This was because, first, in the local context of sago farming, males have a cultural role as the head of family. As such, men have additional responsibility to answering matters related to daily life. Second, local and national officials, most directly involved in handling sago, were predominantly male.

#### Data collection

Each semi-structured interview lasted between 30 and 60 min. Interviews were performed in Indonesian language (Bahasa), and were conducted by the gatekeepers or the lead author. The gatekeepers provided access to several key figures in the local government, community members, and academics with relevant interests. They helped the lead author and conducted twelve interviews. Interviews were either audio-recorded (*n *= 4), recorded in writing (*n *= 10), or recorded on zoom (*n *= 4), and transcribed *verbatim* where applicable.

During the pandemic Covid-19, in-person data collection became problematic due to travel restrictions. Hence, the initial plan for data collection had to change given the partial lockdown in West Papua. Given the social distancing restrictions in place, all data collection took place online via zoom or phone call. However, online data collection was associated with some problems. For example, internet connections in West Papua were not always optimal, and some respondents were reluctant to complete the interview questions in the form of a survey, which made data collection more complex. Communication with the gatekeepers became more challenging with distance, despite the researcher maintaining regular contact through a WhatsApp group. Nonetheless, the range and depth of opinions identified suggests that alternative methods of data collection were rigorous and the results robust.

### Data analysis

A thematic analysis was performed using Nvivo [38] to facilitate data coding, analysis, and organisation, and applied to the interview transcripts. Thematic analysis allows a flexible approach for reporting the perspectives of different research participants and summarising key features of a large dataset [39]. In this research, the meaning unit of analysis was an entire phrase taken from the interviews [40]. The meaning units were labelled with codes that were sorted and collated into themes. A theme in this context represented a group of codes that captured something important about the data in relation to the research questions [41]. The lead author examined the transcripts independently and identified a priori themes based on the research questions, as well as new themes that emerged from the data. These were discussed by the lead author and the rest of the research team until any contradictions were thoroughly checked and themes were established. The final set of themes and illustrative quotes from participants are reported. Quotes have been translated from the local language used (Bahasa Indonesia) in the interviews into English.

## Results

Interviews were collected between March 2020 and June 2021. The main themes and sub-themes identified are presented in Table 2. The number of times the sub-themes mentioned by the stakeholders were coded in descending order are as follows: economic factors (*n *= 10), role of stakeholders in the sago supply chain (*n *= 10), policy and promotion (*n *= 9), cultural identity (*n *= 8), emergency food (*n *= 5), potentially healthy food (*n *= 4), infrastructure (*n *= 4), social and political factors (*n *= 3), price (*n *= 2), qualified human resources (*n *= 2), changes in consumption trends (*n *= 2), and environmental concern (*n *= 1). These are discussed in the remainder of the results section and supported with relevant quotes.

**Table 2:** Themes and sub-themes

Themes	Sub-themes	Description
Motivators to produce and consume sago	Economic factors	a. Sago will provide more financial benefit when processed and sold
		b. Every part of sago is useful
		c. Sago is beneficial for local economy
		d. Sago enables economic relationships with sago companies to be established
	Emergency food	a. Sago provided food stability during Covid-19 pandemic
		b. When food security improves, people choose rice as the first dietary option
	Potentially healthy food	Food to prevent colon cancer and diabetes
	Environmental concern	Heavy machinery used by the sago companies has caused environmental damage
Strategies from stakeholders to increase the awareness of sago consumption	Role of stakeholders in the sago supply chain	a. Government is entirely responsible for sago and its development
		b. Collaboration between community, government, and private sector is required to promote sago
		c. Too many stakeholders involved in sago management is problematic
	Policy and promotion	a. The national government and local government policies are not synchronised
		b. Promotion of sago consumption through social activities and festivities
Barriers to sago consumption	Infrastructure	a. Distance and lack of supporting infrastructure
		b. Sago processing is time and energy consuming
	Price	a. Low selling price
		b. Raskin's (subsidised rice for poor households) policy in remote locations
	Qualified human resources	Lack of qualified human resources
	Social and political factors	a. Sociocultural issues (regionally specific)
		b. Certain parties exploit the issue of sago for their own political gain
Sago eating culture	Cultural identity	Sago is not only as staple food, but is embedded in local cultural practices
	Changes in consumption trends	Rice has become the Papuan people's staple food

### Motivators to produce and consume sago

The main themes that influence the indigenous peoples of West Papua to produce and consume sago were identified; namely economic factors, the use of sago as valuable food in an emergency context, and potential benefits of sago to human health, as well as environmentally beneficial food production. Most stakeholder participants believed that sago has an economic value which benefits the families of those growing it, and/or after the sago has been processed and sold, and which had a positive impact on the local economy.

*Sago has long been a source of revenue for the region. Sago has a high economic value. Derivative products can be a variety of products, from cakes to cosmetics. (participant #01, politician, male).*

The economic value was described in terms of the relation between the local community and the sago processing company in Sorong Selatan.

*Sago may be processed into two economically valuable products: wet and dry sago starch, both of which give benefits to the family (participant #11, sago farmer, male).*

*The economic value of sago starch sold in the market can be used to meet family needs (participant #12, sago farmer, male).*

*During the factory trial, one family meticulously collected sago from their own land and earned 16 million rupiahs in 20 days (participant #18, food activist, male).*

Every part of the sago tree and its derivatives were thought to be economically valuable and beneficial in daily life for most households.

*“… every part of the sago tree has a variety of applications, including roofing, arrows, house walls, and food items, even sago caterpillars may be consumed. Indeed, it is possible to consume the mushrooms that grow on the roots of the sago palm (participant #04, local civil servant, male).*

However, in the context of community interaction with sago companies, some aspects were unfavourable to the community, such as low selling prices and community poverty levels, that require immediate attention.

*In relation to ANJ,*^3^* the low sago selling price remains an impediment and inhumane practice. This is where the government's role in assisting the community comes into play. One sago tree may sustain a household for two months if it is cut down. Ironically, the majority of people with low incomes own sago villages*^4^* (participant #07, academia, female).*

In addition to its economic value, sago was perceived to play a critical role in ensuring food availability at the local level, and, according to stakeholders, particularly during the Covid-19 pandemic. An increasing number of people were relying on sago consumption to achieve their daily nutritional requirements.

*Sago consumption increased significantly during Covid-19, owing to the fact that sago is the easiest crop to obtain without spending money, in comparison to rice (participant #12, sago farmer, male).*

*During the Covid-19 pandemic, sago re-established itself as a staple food, and consumption increased (participant #01, politician, male).*

While sago consumption increased during the pandemic, stakeholders argue that sago consumption will reduce as people's incomes improve.

*When we have money, we eat rice. Without money, sago becomes a viable choice for survival (participant #04, local civil servant, male).*

Two other factors influenced a person's decision to consume sago, namely health and environmental concerns.

*One of the [potential] benefits of sago is that it has a low glycemic index, which helps prevent colon cancer and diabetes. According to my research, if people consume sago or sago rice daily, their health would be preserved (participant #08, academia, male).*

*The sago company is causing havoc on the environment by using heavy machinery without regard for sago and other flora (participant #04, local civil servant, male).*

### Strategies from stakeholders to increase the awareness of sago consumption

Given that stakeholders generally supported maintaining or increasing sago consumption within the local population, primarily to promote food security in relation to food availability and nutrition, it follows that strategies may be needed to enhance public awareness about the potential benefits of sago consumption. The majority of stakeholder participants believed that the government is primarily responsible for sago consumption promotion.

*The government is responsible for sago (participant #01, politician, male).*

*Local governments must take a proactive role in safeguarding community rights regarding sago forests (participant #02, local civil servant, male).*

However, it was thought that sole dependence on the government to support sago production and consumption within local diets, would not be effective. Effective collaboration between the community, government, and private sector was thought to be needed to jointly increase public awareness of the importance of sago in everyday life.

*The community and local government are responsible stakeholders (participant #05, local civil servant, male).*

*If people are not accustomed to eating sago, no matter how strict the government's local laws are, they will fail. We must first love what we have. Akness,*^5^* ANJ, and Perhutani*^6^* are the backbones of Sorong Selatan, as no one else in Papua is capable of managing sago as well as we are (participant #07, academia, female).*

Whilst involving different stakeholders in sago management was thought to be important by stakeholders, participants expressed the view that having too many stakeholders involved in policy development and the supply chain may also be problematic, potentially because this would slow the policy process down because of lack of consensus across stakeholder groups. It should be noted that, the existing literature on stakeholder engagement does not support this contention.

*One of the challenges that has existed in the past and continues to exist today is that there are too many agencies that handle it, resulting in an underdeveloped sago [value chain] (participant #08, academia, male).*

After gaining a thorough understanding of stakeholder priorities and perspectives an effective policy and promotion plan is required. As a result, policy synergy between the national government and local governments is necessary. Yet the national government and local government policies are not synchronised.

*The government is involved in fostering the development of the infrastructure as part of national strategy. Road infrastructure in order to make distribution lines more efficient. Additionally, the regional government develops regional regulations governing the price of sago (participant #01, politician, male).*

*Until today, Sorong Selatan has lacked a dedicated sago policy, despite the advantages of the sago hectare area (participant #05, local civil servant, male).*

*There is no local legislation governing, for example, the distribution of rice and sago to employees, where out of 10 kg of rice, 3 kg is sago (participant #07, academia, female).*

In practice, sago promotional activities carried out by local and national government continue to be limited to ceremonial occasions, such as social events and food festivals.

*The local government's policy in terms of promoting sago is limited to festivals that promote sago culture/traditions (participant #02, local civil servant, male).*

*Social events, courses, and instruction on how to prepare sago-based foods (participant #03, local civil servant, male).*

*A call from West Papua's province administration that whenever there is an activity, local food options such as sweet potatoes, taro, bananas, and sago must be provided, including for families and in churches (participant #07, academia, female).*

### Barriers to consume sago

Participants believed that there are numerous difficulties and challenges in the area of sago (production) management that affect sago consumption. One obstacle is the distance to and a lack of supporting infrastructure for farmers to go to the sago forest, not to mention the sago processing which requires considerable time and energy from those involved.

*Apart from the manual process, I believe that the distance to the sago location is one of the problems encountered (participant #03, local civil servant, male).*

*Obstacles related to the harvesting process which requires close proximity to rivers for transportation of harvested sago (participant #12, sago farmer, male).*

*Processing sago into starch involves a great deal of labour and time, making this activity tough (participant #01, politician, male).*

*Sago rice took a long time to develop [research and development process]; for me, it took 20 years for sago to be processed into the sago rice that exists today (participant #08, academia, male).*

After harvesting sago, an additional problem may be the unfavourable selling prices. Sago has a low selling value, especially if compared to the efforts expended in the harvesting process which takes a long time and is labour intensive. In addition, Raskin's policy (subsidised rice for poor households) has been applied to households in remote locations. People therefore prefer to consume rice rather than sago, given its convenience and accessibility.

*The market practice of purchasing and selling sago continues to be destructive to us. For instance, the price of a single stick of Surya cigarette [one of the local cigarette brands] is higher than the price of sago. This makes no sense at all (participant #04, local civil servant, male).*

*Raskin’s policy [subsidised rice for poor households] has applied to remote locations, and families and farmers have lost interest in planting or cultivating sago in the forest (participant #01, politician, male).*

The lack of skills in relation to sago processing has contributed to sago's underdevelopment as a commodity. The two main issues in terms of resources are the inaccessibility of technical equipment and trained value chain workers who can manage sago from planting to harvesting.

*The difficulty is in obtaining the necessary equipment for sago production, but the government has aided in this endeavour (participant #05, local civil servant, male).*

*In terms of sago cultivation, the community still lacks the competence necessary to manage the crop using qualified practices, from seedling to harvest (participant #07, academia, female).*

Finally, managing sago cultivation is not just a technical concern; it also requires motivation on the part of farmers and producers to engage in, and manage, production effectively. One of the socio-cultural aspects that hinder the development of sago is related to customary land ownership in the forest owned by the indigenous people, as well as the perceived problem of a relatively low work ethic. Stakeholders recognise that there is a need to ensure (economic and agricultural) policies are linked to sago production, and that indigenous people are engaged in the policy (and sago production) processes. For example, contributing local knowledge, skills and expertise complement those held by workers from outside the area.

*The issue with sago development is not technical, but rather socio-cultural (participant #16, national civil servant, male).*

*The difficulty arises solely from a lack of will to manage the sago (participant #18, food activist*, male).

*Economic management is still closely linked to the political process in relation to sago. When a large number of the workers are from outside Papua, the situation becomes political. Indigenous Papuans are merely observers, despite the fact that investment also requires specific skills and expertise (participant #14, national civil servant, male).*

### Sago eating culture

The majority of participants agreed that sago is embedded in a cultural and ancestral identity that must be protected, and indicated that at present, the visible form of preserving sago eating culture has become largely limited to cultural and food festivals. Traditional food may be included in diets more generally and also contributes to cultural identities.

*Sago continues to play a significant role today, employs the indigenous system, and is an ancestral tradition. Sago is a defining feature of the Papuan people that cannot be lost across generations (participant #02, local civil servant, male).*

*Sago Papeda*^7^* has been my staple diet from childhood, passed down from generation to generation, enabling me to attend school, read, and write. When sago is not available, the Papuan people perish, as our existence is dependent on sago (participant #04, local civil servant, male).*

*Sago is the primary diet of the Papuan people, and we have known it since we were born (participant #13, sago farmer, male).*

Consumption trends, however, appear to have changed, potentially as a consequence of various other factors, such as the increased availability and accessibility of rice (compared to sago), and policies which subsidise rice for consumption in poorer households (i.e. Raskin’s policy), resulting in rice having become the Papuan people's staple food.

*When we discuss staple foods, we will remark that sago is the Papuan people's staple food. However, if we look at daily life, we see that rice is the staple food that has developed into its own lifestyle. This is in contrast to the rural areas, where sago is more readily available for consumption (participant #07, academia, female).*

*When it comes to the Papuan people's food, rice remains the primary staple, followed by sago (participant #01, politician, male).*

## Discussion

The aim of this qualitative case study was to understand the perspective of a selected number of different stakeholders regarding sago consumption in terms of both agronomic practices and dietary choices. The results indicate that stakeholders consider that the majority of the Papuan people regard sago as a traditional food that is important to, and inseparable from, every aspect of their lives, although this requires further confirmation with Papuan consumers themselves. Although sago has long been known as the staple food of the Papuan people, holding a special place in Papuan culture as one of the prerequisites for certain tribal traditional ceremonies [42], there is evidence from stakeholders that sago consumption within the general diet of Papuans is declining. This reduces the resilience of local food systems to system shocks as there is increased reliance on imported foods. In addition, other aspects of sago production and consumption require further consideration. For example, these results suggest that sago contributes to the economic well-being of local farmers by increasing their income, promoting the local circular economy, through reuse of sago residues, which reduces the polluting effects from sago processing industries, and also provides an economic solution for waste management systems [43, 44]. Sago farming also contributes to the maintenance or restoration of local ecological systems [45]. The results confirm previous research findings which indicate that indigenous foods have the potential to promote food security, including during times of crisis such as the Covid-19 pandemic [46]. In Papua, stakeholders perceive that this is because sago is a food source that is locally grown and adapted to local agronomic and climatic conditions, which has potential to significantly reduce the Papuan people's reliance on imported food supplies, such as in the case of the Covid-19 pandemic which has disrupted the food supply chain.

All of the stakeholders participating in this research expressed the view that sago is a healthy food. However, environmental issues were not a primary focus of stakeholders’ interest. Indeed, discussion was limited to the negative environmental impacts of sago production, for example, in relation to the sago companies that harm the environment during the production process, such as harvesting sago without regard for the surrounding. This particular situation requires immediate remediation. However, the environmental profile of sago production is relatively positive. For example, recent research indicates that the greenhouse gas (GHG) emissions associated with producing 1 tonne of sago (17.9 kgCO_2_eq) are significantly less than those associated with corn starch (2700 kgCO_2_eq), potato starch (2402 kgCO_2_eq), and cassava starch (4310 kgCO_2_eq) [47]. This means that sago production has strategic value in terms of reducing the local negative environmental impacts of agriculture, especially when compared to the palm forestations.

In the context of national policy, it is important to consider sago production based on Law Number 2 of 2021 concerning the Second Amendment to Law Number 21 of 2001 concerning Special Autonomy for the Province of Papua. The main objectives of this law are to improve the quality of public services as well as sustainable development in the Papua region. This is also supported by the establishment of the Papuan People's Assembly (MRP) which is the cultural representation of the indigenous peoples of Papua who have certain authorities in the context of protecting the rights of the indigenous peoples based on respect for customs and culture, empowerment of women, and ensuring a harmonious religious life. In the context of sago management, the ambition is that existing regulations at the national level can also be implemented properly with local regulations in the regions. This study demonstrates that the local government has not yet established comprehensive sago management policies for the community's benefit.

In addition, there is a disagreement between academics and local government in terms of whether or not to improve the quality of human resources in sago management through the education sector. Academic stakeholders expressed the view that the local government should pay more attention to Akness as the only sago academy in West Papua that focuses on developing sago as a leading commodity, improving the effectiveness of extension services and policies. Regardless of the differing political agendas of each regional leader, local government must immediately prioritise the education sector, particularly sago in whatever shape it takes. It is anticipated that the synergy between the local administration and academics will enhance support for initiatives to restore sago as a cultural asset of the Papuan people that must be protected.

Some of the stakeholders interviewed were civil servants with a direct influence on policy development and implementation. The engagement of other stakeholders (e.g. representatives of civil society) is recognised as being an important part of the development of policy [48]. Taken together, the results have provided evidence which will help understanding of the barriers to, and facilitators of, increased production and consumption of sago in the regions included in the research, and potentially within a broader geographic area.

Stakeholders emphasised the important roles of local government in promoting sago production and consumption. There is stakeholder awareness that local government policies to promote sago production will not be successfully implemented without the engagement of local communities in relation to sago production, inclusion in diets, and as part of the cultural wealth of the Papuan and Indonesian people. At the same time, stakeholders recognised that the community wants clearer rules for professional sago management efforts. Therefore, the collaboration of indigenous peoples and local governments is very important to consider within policy development and implementation, especially as regional autonomy prioritises regional independence and innovation. Currently, although policies at the national level have provided general guidelines to preserve cultural tradition, local governments need to formulate policies that are able to accommodate specificities of particular traditions at the local level. The policy must be formulated to anticipate the various obstacles that exist locally, including addressing barriers to sago production and consumption, such as the introduction of more efficient and less time consuming sago production practices, and policies which encourage consumption by local people.

The effectiveness of developing such an approach is illustrated by recent developments in Sorong Selatan which demonstrate the strong synergy between indigenous peoples and local government. The regent of Sorong Selatan is currently facing lawsuits from two palm oil companies whose licenses have been revoked [49]. The revocation of this business licence is based on an assessment of the licencing policy conducted by the West Papua province and the local government, as well as indigenous peoples' aspirations for welfare, safety, and the long-term viability of the environment's sustainability. This illustrates how the conversion of sago forest into oil palm plantations has become a new problem for indigenous peoples in Sorong Selatan.

This research represents the first qualitative research, to our knowledge, that has examined stakeholder’s perceptions of the motivators, strategies, and barriers to sago consumption in West Papua, Indonesia. We were able to engage participants in rich discussion relevant to the research questions by using semi-structured interviews. Furthermore, the research adhered to COREQ's guidelines for conducting and reporting qualitative research, demonstrating rigour in data collection and analysis. However, the research has a number of limitations. The small sample size, which has resulted in only a few stakeholders in each stakeholder category being represented means that the results may not have reached “saturation” where no additional information is being identified. The lack of female participants, a consequence of the gatekeepers’ limited ability to facilitate their recruitment, may have introduced a gender-related bias to the interpretation of the results. The data quality *may* have been richer had personal face-to-face interviews been conducted, which reflects the difficulty of collecting interview during the Covid-19 pandemic. Finally, while generalisation is not a goal of qualitative research, our findings should be interpreted within the context of the data collection, which consisted of a small sample of participants from a single geographic area. As a result of the limitations of the studies in this research area, future studies should be considered whether consumer studies align with stakeholder views regarding sago consumption. Future research might also consider using alternative approaches to understanding stakeholder, consumer and other supply chain actor perspectives combining qualitative, quantitative and ethnographic approaches, which will allow for the triangulation of results to further inform the research question. Further research into the sago food system might bring in expertise in biodiversity, human nutrition and economics, which will address environmental, nutritional and fiscal perspectives using appropriate methodologies.

## Conclusion

The evidence presented here suggests a diverse range of local stakeholders support the continuation of sago production and consumption, both from the perspective of improved food security, and in order to conserve cultural associations and activities within local communities. According to the stakeholders interviewed, this can be best achieved through improved engagement of local communities with sago production policies, innovation in current practices, and agronomic management. Local policies should be developed to ensure that sago remains an integral component of the Papuan people's culture, and develops into a significant commodity with economic value which simultaneously contributes to environmental targets. The results indicate that local stakeholders are positive about the inclusion of sago in the diets of indigenous people, not only to improve food security in West Papua, but also as a foundation for developing future food security policies.

## Supplementary Information

**Additional file 1.** COREQ checklist.

## Data Availability

The data of this study are available upon request from the authors.

## Footnotes

1 Artificial rice is non-paddy rice that looks like rice and is made from high-carbohydrate local resources such as potatoes, cassava, corn, and sago.

2 Two gatekeepers were involved in the research: a civil servant in the Sorong Selatan Regency and an environmental activist.

3 PT ANJ Agri Papua Barat (ANJAP) is one of the sago companies in West Papua operating since September 2007.

4 Sago villages is a forest that is naturally overgrown by sago, inherited based on customary law by their respective parents in each tribe, and managed from generation to generation.

5 Akness (Akademi Komunitas Negeri Sorong Selatan) is a vocational education institution based on the Community Academy which was established to prepare human resources in Sorong Selatan for the development of sago in the future.

6 Perhutani is a state forest enterprise that established a sago factory in Sorong Selatan.

7 Papeda is a traditional Indonesian dish made with sago that is frequently referred to as the local porridge variety for the indigenous peoples of Papua and Maluku.
