# Supplementary material for: Factors influencing consumption of traditional diets: stakeholder views regarding sago consumption among the indigenous peoples of West Papua
Source: Agric Food Secur. 2022 Oct 7;11(1):51. doi: 10.1186/s40066-022-00390-5 (PMC9540296; doi:10.1186/s40066-022-00390-5)
Supplement: Supplementary file 1 — Additional file 1. COREQ checklist. [file 40066_2022_390_MOESM1_ESM.pdf]

Manuscript: Factors Influencing Consumption of Traditional Diets: Stakeholder Views regarding Sago Consumption among the Indigenous Peoples of West Papua.

Consolidated criteria for reporting qualitative studies (COREQ): 32-item checklist

Developed from:
Tong A, Sainsbury P, Craig J. Consolidated criteria for reporting qualitative research (COREQ): a 32-item checklist for interviews and focus groups. *International Journal for Quality in Health Care*. 2007. Volume 19, Number 6: pp. 349 – 357

**No. Item**	**Reported on #**
**Domain 1: Research team and reflexivity**	
Personal Characteristics	
1. Interviewer/facilitator<br>2. Credentials<br>3. Occupation<br>4. Gender<br>5. Experience and training	**Fathir Fajar Sidiq** is a PhD student at School of Natural and Environmental Sciences, Newcastle University. He is interested in using mixed methods to investigate food policy in Indonesia, particularly sago as traditional food for the indigenous peoples of Papua.<br><br>**David Coles** whose background is in chemistry and ethics, is a Senior Research Fellow at the Centre for Professional Ethics, UCLan, a Research Associate at the University of Newcastle's School of Agriculture, Food and Rural Development and Director of Enhance International Ltd. His interests include ethical and risk communication issues, particularly in relation to food production and security, biomedical ethics, multidisciplinary, converging and emerging technology research, innovation and the relationship between science, technology and policy.<br><br>**Carmen Hubbard** is a senior lecturer in Center for Rural Economy at School of Natural and Environmental Sciences, Newcastle University. She is a quantitative researcher with the area of expertise in agricultural economics focusing on the economics and political economy of Common Agricultural Policy (CAP), agriculture and rural development, farm animal welfare, applied welfare economics, and comparative country analysis.

	**Beth Clark** is a research associate at School of Natural and Environmental Sciences, Newcastle University. Her research interests include exploring the use of mixed and evidence-based methods in exploring public perceptions towards animal welfare and meat consumption, specifically qualitative and quantitative systematic reviews, and mixed qualitative research methods.<br><br>**Lynn J. Frewer** is a professor of food and society at School of Natural and Environmental Sciences, Newcastle University. She has interests in all areas of food and society, including those areas which require transdisciplinary collaboration between the social and natural sciences.
Relationship with participants	
6. Relationship established	Page 3
7. Participant knowledge of the interviewer	Page 3
8. Interviewer characteristics	See above

**Domain 2: study design**	
Theoretical framework	
9. Methodological orientation and Theory	Page 3
Participant selection	
10. Sampling	Page 3
11. Method of approach	Page 3
12. Sample size	Page 3
13. Non-participation	N/A
Setting	
14. Setting of data collection	Page 4
15. Presence of non-participants	Page 4
16. Description of sample	Page 4
Data collection	
17. Interview guide	Page 4
18. Repeat interviews	N/A
19. Audio/visual recording	Page 4
20. Field notes	Page 4
21. Duration	Page 4
22. Data saturation	Page 4, 10
23. Transcripts returned	N/A
**Domain 3: analysis and findings**	
Data analysis	
24. Number of data coders	Page 5
25. Description of the coding tree	N/A
26. Derivation of themes	Page 5
27. Software	Page 5
28. Participant checking	N/A
Reporting	
29. Quotations presented	Page 6 – Page 9
30. Data and findings consistent	Page 9 – Page 10
31. Clarity of major themes	Page 9 – Page 10
32. Clarity of minor themes	Page 10
